# Disequilibrium between *BRCA1* and *BRCA2* Circular and Messenger RNAs Plays a Role in Breast Cancer

**DOI:** 10.3390/cancers15072176

**Published:** 2023-04-06

**Authors:** Corentin Levacher, Mathieu Viennot, Aurélie Drouet, Ludivine Beaussire, Sophie Coutant, Jean-Christophe Théry, Stéphanie Baert-Desurmont, Marick Laé, Philippe Ruminy, Claude Houdayer

**Affiliations:** 1 Univ Rouen Normandie, INSERM U1245, FHU-G4 Génomique, 76000 Rouen, France; corentin.levacher@univ-rouen.fr (C.L.);; 2 Univ Rouen Normandie, INSERM U1245, Centre Henri Becquerel, 76000 Rouen, Francemarick.lae@chb.unicancer.fr (M.L.); philippe.ruminy@chb.unicancer.fr (P.R.); 3 Department of Pathology, Centre Henri Becquerel, 1 Rue d’Amiens, 76038 Rouen, France; 4 Department of Medical Oncology, Centre Henri Becquerel, 1 Rue d’Amiens, 76038 Rouen, France; 5 Univ Rouen Normandie, INSERM U1245, FHU-G4 Génomique and CHU Rouen, Department of Genetics, 76000 Rouen, France

**Keywords:** breast cancer, circular/messenger RNAs, *BRCA1*/*BRCA2*

## Abstract

**Simple Summary:**

Breast cancer is a common disease that is responsible for the deaths of approximately 700,000 women per year i.e., 15.5% of all female deaths from cancer. Significant progress has been made in the management of breast cancer but new biomarkers for early detection and prognosis have yet to be identified. Circular RNAs are a class of transcripts that are still poorly understood and share their mode of production with messenger RNAs. We have studied the circular RNA/messenger RNA pair in *BRCA1*- and *BRCA2*-related breast tumorigenesis using an innovative dedicated technique and have shown for the first time that there is an imbalance in the circular RNA/messenger RNA ratio between healthy and tumor breast tissues. This very new observation in breast cancer opens up new perspectives in terms of new mechanisms and the associated biomarkers.

**Abstract:**

Breast cancer is a frequent disease for which the discovery of markers that enable early detection or prognostic assessment remains challenging. Circular RNAs (circRNAs) are single-stranded structures in closed loops that are produced by backsplicing. CircRNA and messenger RNA (mRNA) are generated co-transcriptionally, and backsplicing and linear splicing compete against each other. As mRNAs are key players in tumorigenesis, we hypothesize that a disruption of the balance between circRNAs and mRNAs could promote breast cancer. Hence, we developed an assay for a simultaneous study of circRNAs and mRNAs, which we have called splice and expression analyses by exon ligation and high-throughput sequencing (SEALigHTS). Following SEALigHTS validation for *BRCA1* and *BRCA2*, our hypothesis was tested using an independent research set of 95 pairs from tumor and adjacent normal breast tissues. In this research set, ratios of *BRCA1* and *BRCA2* circRNAs/mRNAs were significantly lower in the tumor breast tissue compared to normal tissue (*p* = 1.6 × 10^−9^ and *p* = 4.4 × 10^−5^ for *BRCA1* and *BRCA2*, respectively). Overall, we developed an innovative method to study linear splicing and backsplicing, described the repertoire of *BRCA1* and *BRCA2* circRNAs, including 15 novel ones, and showed for the first time that a disequilibrium between *BRCA1* and *BRCA2* circRNAs and mRNAs plays a role in breast cancer.

## 1. Introduction

Breast cancer (BC) represents one-quarter of all cancers in women and ranks first in terms of incidence among female cancers (https://gco.iarc.fr, accessed on 15 March 2023) [1]. According to recent data from Global Cancer Statistics 2020, the deaths of almost 700,000 women per year (15.5% of all female deaths by cancer) are caused by BC [1]. BC is a heterogeneous disease with specific molecular, histological, and clinical features. Despite effective treatments, the need for new genetic markers to enable early detection or prognostic assessment remains. Recently, circular RNAs (circRNAs) have emerged as a promising field of study. CircRNAs are single-stranded covalent structures in closed loops without a 3′ cap or a 5′ poly-A tail, which are produced by backsplicing i.e., the junction of a donor site to an acceptor site located upstream [2] (Figure S1 in the Supplementary Materials). CircRNAs contain complete exons of protein-coding genes, can be transcribed by the RNA polymerase II, and are mediated by the spliceosome. This circularization protects them from degradation by RNA exonucleases, their half-life thus being longer than their linear counterparts [3].

The expression of circRNAs is tissue- and cell-type-specific [4,5], and several elusive roles have been described, such as (i) microRNA (miRNA) sponges [6], (ii) RNA binding protein sponges [7], or (iii) modulators of transcription [8]. In any case, previous work supports the implication of circRNAs in cell proliferation, apoptosis, or metastasis [7,9,10]. With regard to BC, whole transcriptome-based studies showed the deregulation of specific circular RNAs, pinpointing their role as miRNA sponges within the frame of circRNA-miRNA-mRNA networks [10].

Given that circular RNA and mRNA are generated co-transcriptionally [11] and that the amount of circRNA can exceed the amount of mRNA of the host gene [12], we believed that circRNA may have another, more pronounced impact on tumorigenesis by the direct deregulation of mRNA levels. The involvement of mRNAs in cancer is indeed well-documented; e.g., alternative splicing alterations are known to affect epithelial-mesenchymal transition, apoptosis, and cell proliferation [13,14,15,16]. Moreover, pathogenic variations impacting splicing could represent the most frequent class of alterations seen in cancer [17]. Thus, any other class of transcripts that could impact mRNAs could influence the tumor phenotype; circRNA biogenesis has been precisely described as competing with pre-mRNA splicing in a muscle-blind gene model [11]. We hypothesized that proper gene regulation could mandate a balance between both transcripts, therefore balance disruption between circRNAs and mRNAs could promote tumorigenesis. To test this hypothesis, we used breast cancer samples and the *BRCA1* and *BRCA2* genes as a model. To study messenger RNAs and circular RNAs without the complexity and high costs of transcriptomic RNA-seq approaches, we developed an alternative, innovative high-throughput, and gene-targeted approach, known as splicing and expression analyses by exon ligation and high-throughput sequencing (SEALigHTS). SEALigHTS allows the simultaneous study of splicing and backsplicing for *BRCA1* and *BRCA2* and measures the balance of circRNAs/mRNAs.

We have characterized novel *BRCA1* and *BRCA2* circular RNAs and, for the first time, described a disequilibrium in circRNA/mRNA levels between tumor and normal breast tissues.

## 2. Materials and Methods

### 2.1. Patients

**Figure 1:**
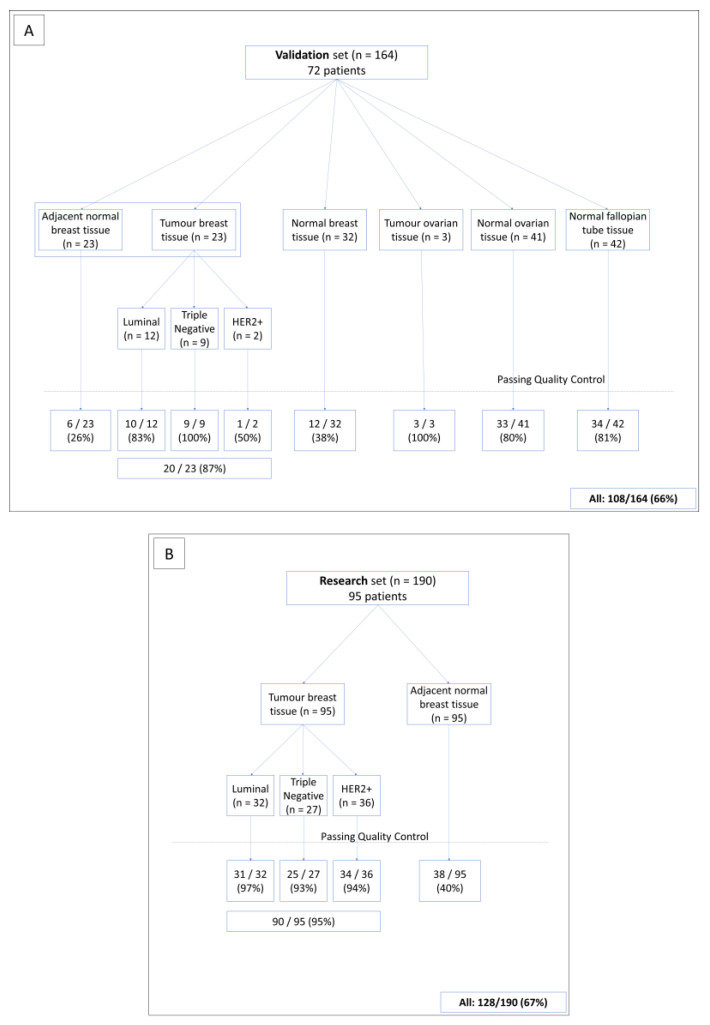
Description of the validation set (**A**) and the research set (**B**). The quality control corresponds to the number of UMI above 1500 for a given sample.

SEALigHTS was first validated on a formalin-fixed, paraffin-embedded (FFPE) set of samples from 72 selected female patients carrying germline pathogenic variations (PV) on *BRCA1* or *BRCA2*, collected between 2017 and 2020. Written informed consent was obtained for all patients who were tested and diagnosed within the frame of genetic counseling. Hence, 164 FFPE tissue samples were obtained from these 72 patients, consisting of 23 pairs of tumor samples (invasive breast carcinomas consisting of all BC subtypes (12 luminal, 2 HER2-positive, and 9 triple-negative breast carcinomas (TNBCs)) and normal adjacent breast tissue samples, 32 normal mammary tissue samples deriving from prophylactic mastectomy, 3 tumor tissue samples from serous invasive carcinoma, 41 normal ovarian tissue samples from prophylactic oophorectomy, and 42 normal tissue samples from fallopian tubes (Figure 1A). All samples were reviewed by an expert pathologist to confirm the diagnoses and evaluate breast cancer subtypes according to the following recommendations. Cases were designated estrogen (ER)- or progesterone (PR)-negative, according to the French national criteria, if less than 10% of the tumor cells expressed ER/PR [18]. Immunohistochemistry (IHC) was used to evaluate HER2 expression, with grading based on the American Society of Clinical Oncology (ASCO)/College of American Pathologists (CAP) criteria [19]. BC subtypes were defined as follows: tumors testing positive for either ER or PR and negative for HER2 were classified as luminal; tumors testing positive for HER2 were considered to be HER2-positive BC; tumors testing negative for ER, PR, and HER2 were considered to be triple-negative BC (TNBC). Nine PV, identified in 15 patients, were likely to impact *BRCA1* or *BRCA2* splicing, i.e., 4 single nucleotide variations (SNV), 3 large deletions, and 2 large tandem duplications (Table S1).

Following SEALigHTS validation, 190 normal and tumor breast tissues from 95 unselected, consecutively ascertained patients without genetic evaluation (i.e., the patients were not referred to the genetics clinics) were analyzed in a research phase to test our hypothesis (Figure 1B). Overall, 354 FFPE tissue samples were analyzed. All FFPE tissues were retrieved from the biobank of the Henri Becquerel Cancer Center. Written informed consent was obtained for all patients.

### 2.2. RNA Extraction

One H&E-stained slide from formalin-fixed paraffin-embedded tissue (FFPE) was obtained for each sample and reviewed by an expert pathologist to evaluate tumor cellularity, which was always greater than 15%. RNA was then isolated from 8 consecutive 10 µm unstained slides, using the automated Maxwell^®^16 research extraction system (Promega, Madison, WI, USA) and the Maxwell^®^16 FFPE Plus LEV RNA Purification Kit following the manufacturer’s instructions, and was stored at −80 °C. An RNA concentration evaluation was performed using the Qubit fluorometer (Invitrogen, Carlsbad, CA, USA).

### 2.3. SEALigHTS Assay

#### 2.3.1. Principle

**Figure 2:**
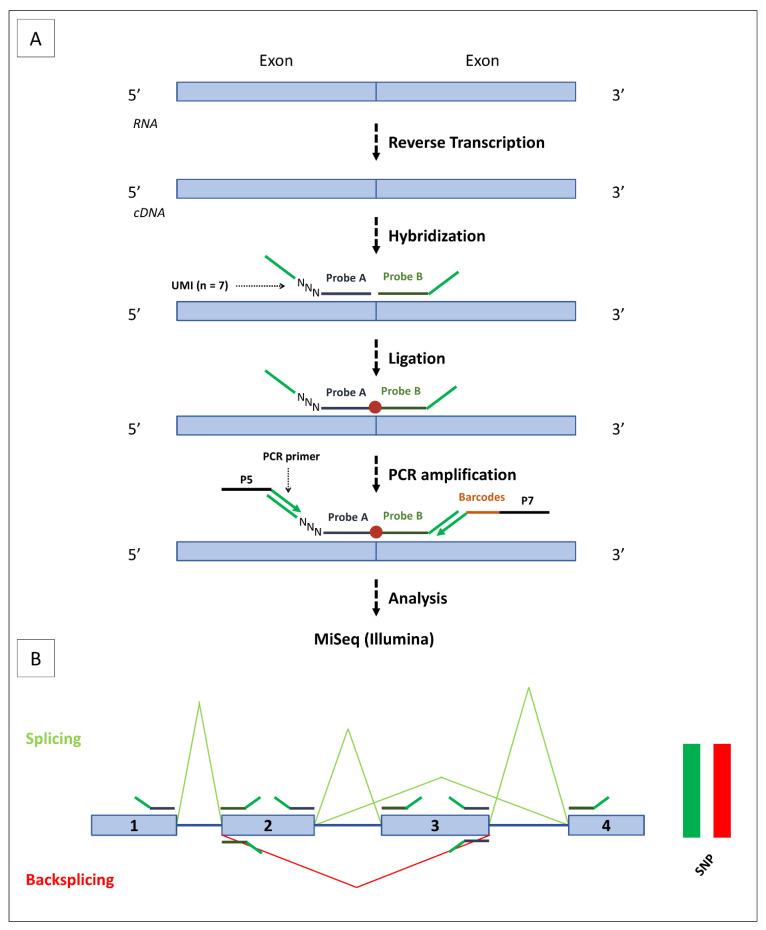
SEALigHTS assay. (**A**) Following cDNA synthesis, the probes are hybridized at exon extremities. Left probes include UMI consisting of a stretch of 7 random bases, depicted here as NNN. If hybridization takes place, probe ligation (depicted as a red dot) will occur. Then, PCR amplification will be performed using 2 universal complementary primers on the ends of each probe. Primers contain P5 or P7 adapters, which hybridize to the flow cell. Sample identification barcodes are included in the right primer. (**B**) Schematic representation of splicing and backsplicing results. Exons are represented as boxes and are numbered, with introns represented as lines. The probes are placed at exon extremities. Splicing junctions are represented in green, with backsplicing junctions in red. Peak heights depend on the number of UMI counts. Exon 3 skipping and exon 3-exon 2 backsplicing are shown as examples. On the right side, a balanced heterozygous SNP (i.e., with the same number of UMI counts) is depicted in red and green for the 2 allelic versions.

The SEALigHTS assay derives from RT-MLPseq [20] and LD-RTPCR [21], used to measure gene expression and detect fusion transcripts [22,23,24], and was adapted to study splicing and backsplicing simultaneously. Briefly, probes designed at exon boundaries are joined when splicing occurs, then ligated, and the resulting fragment is detected and quantified with a high-throughput sequencer (Figure 2A). All possible combinations of exons and their resulting transcripts, i.e., splicing and backsplicing, are amenable to detection and quantification. To search for allelic imbalance as an indirect consequence of nonsense-mediated decay (NMD), probes targeting the 2 allelic versions of exonic single nucleotide polymorphisms (SNPs) were designed. Lastly, DNA contaminants were evaluated using intronic probes.

#### 2.3.2. Protocol

The oligonucleotide probes contained (i) specific sequences for each exon (20–30 bases in length to obtain an optimal melting temperature of 70 °C), (ii) unique molecular identifiers (UMI), consisting of 7 random bases, to count the number of ligations (Figure 2A). To study splicing and backsplicing, 114 probes were designed at exon boundaries for all *BRCA1* and *BRCA2* isoforms, as described by Davy and co-workers [25], and in the RJunBase, a web-accessible database of three types of RNA splice junctions (linear, backsplice, and fusion junctions) derived from the RNA-seq data of non-cancerous and cancerous tissues [26]. To identify NMD, we designed 30 probes assaying 6 and 4 SNPs on *BRCA1* and *BRCA2*, respectively. To control for DNA contamination, 3 intronic *BRCA1* probes were added to the probe mix (data available on request).

Following quantification, 0.2−600 ng of total RNA were converted into cDNA using a SuperScript™ VILO™ cDNA Synthesis Kit (Invitrogen, Carlsbad, CA, USA). cDNAs were incubated for 1 h at 60 °C with our mix of 147 oligonucleotides probes in 1× SALSA MLPA buffer (MRC Holland, Amsterdam, The Netherlands), ligated using the thermostable SALSA DNA ligase (MRC Holland, Amsterdam, The Netherlands) and amplified using barcoded primers containing P5 and P7 adaptor sequences with the Q5 High-Fidelity 2× Master Mix (NEB, Ipswich, MA, USA). Amplification products were purified using AMPure XP beads (Beckman Coulter, Brea, CA, USA) and analyzed using a MiSeq sequencer (Illumina, San Diego, CA, USA).

The reads are thus composed of at least 150 bases, including the UMI, the left and right probes, and the barcodes introduced during PCR amplification. Sequencing reads are demultiplexed using the barcodes, aligned with the sequences of the probes, and exon junctions are counted. Quantitation is achieved thanks to the 7 random bases of the UMI, allowing 16,384 different combinations of unique molecules. Schematic backsplicing and splicing profiles were generated with a homemade Python script, available on request (Figure 2B).

### 2.4. Ratio of circRNAs/mRNAs

Linear messenger RNAs (splicing) were distinguished from circular RNAs (backsplicing) thanks to the order of the probes. If a probe located at the 3′ boundary of an exon is ligated with a probe located at the 5′ boundary of the following exon, the transcript is linear. On the other hand, if this 3′ probe is ligated to a 5′ probe of a preceding exon, the transcript is circular (Figure 2B). The relative proportion of a single circRNA on a gene is obtained by dividing the number of UMI of this circular RNA by the sum of UMI for every circular RNA.

For each circular RNA, the circRNA/mRNA ratio is calculated by dividing the number of UMI of its circular junction by the average number of UMI of a linear junction of the gene:

(UMI circular junction)/((∑ UMI of linear junctions)/(number of linear canonical junctions))(1)

where the overall circRNA/mRNA ratio is calculated by dividing the number of UMIs of all circular junctions by the number of UMI of all linear junctions of a gene:

(UMI all circular junctions)/(UMI all linear junctions).(2)

## 3. Results

The first step was to validate the SEALigHTS assay for the simultaneous detection of splicing and backsplicing, before embarking upon the research phase.

### 3.1. General Characteristics of the SEALigHTS Assay

The results were interpreted when at least 1500 different UMI per sample were counted for both *BRCA1* and *BRCA2*. The reason for this is that all known alternative transcripts above 1% were detected using this threshold. A total of 354 FFPE samples of various tissue types were analyzed and 236 out of 354 samples (66.5%) passed the 1500 UMI threshold with an important disparity according to the type of sample (Figure 1A). Of the breast tumor tissues in the validation set, 20 out of 23 samples (86%) met the quality criteria, compared to 6 out of the 23 (26%) samples from adjacent normal tissues. Of the prophylactic mastectomy tissues, 12 out of 32 samples (37.5%) passed the threshold, whereas the values were 33 out of 41 (80.5%) for normal ovarian tissue samples and 34 out of 42 (81%) for normal salpingian tissue samples. Overall, 108 samples were retained for the validation set. Similarly, for the breast tissues in the research set (Figure 1B), 90 out of the 95 tumor samples (94.7%) and 38 of the 95 normal tissue samples (40%) generated more than 1500 UMI, respectively. Thus, 128 samples were available for the test set.

### 3.2. SEALigHTS Assay Validation

#### 3.2.1. Splicing Analyses

**Figure 3:**
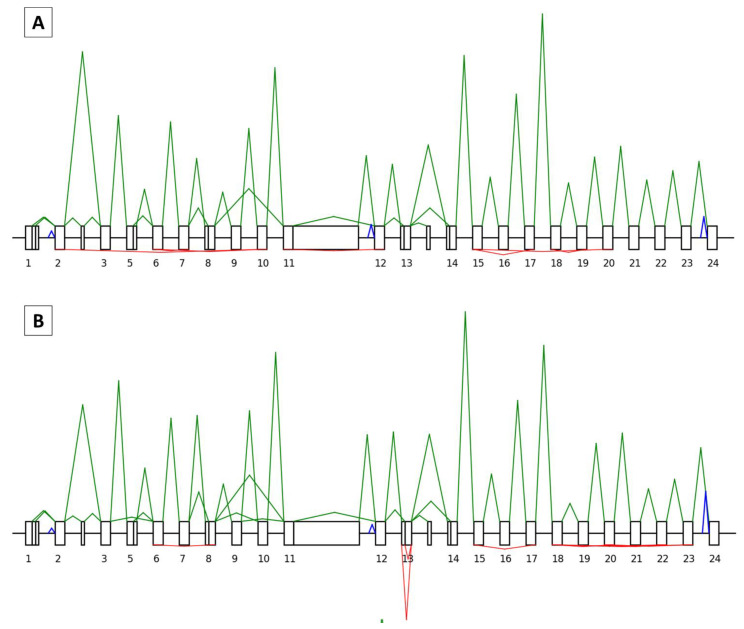
Representation of the *BRCA1* splicing and backsplicing profiles. Exons are drawn as boxes and are numbered, with the introns represented as lines. A solid line indicates alternative splice sites within exons. Non-numbered boxes correspond to alternative exons not included in the canonical transcript. Peak heights are relative to the number of UMI counts. For each profile, splicing is indicated in green above the boxes and backsplicing in red below the boxes. The blue peaks correspond to the signals of the intronic probes. (**A**) Control profile (patient 20X0160, normal breast tissue): the *BRCA1* canonical transcript with different isoforms (delta 5q, delta 8p, delta 9–10, delta 11q, delta 13p, delta 14q) is shown. In red, below, is the backsplicing from exons 17 to 15 (circRNA_17-15), 19 to 18 (circRNA_19-18), 20 to 15 (circRNA_20-15), 7 to 6 (circRNA_7-6), 10 to 2 (circRNA_10-2), and 10 to 6 (circRNA_10-6). (**B**) Tandem duplication of exon 13 (patient 20X0146, tumor breast tissue) is detected thanks to the two pseudo circular RNAs of exon 13 (both the canonical and alternative 13p transcripts are produced).

**Figure 4:**
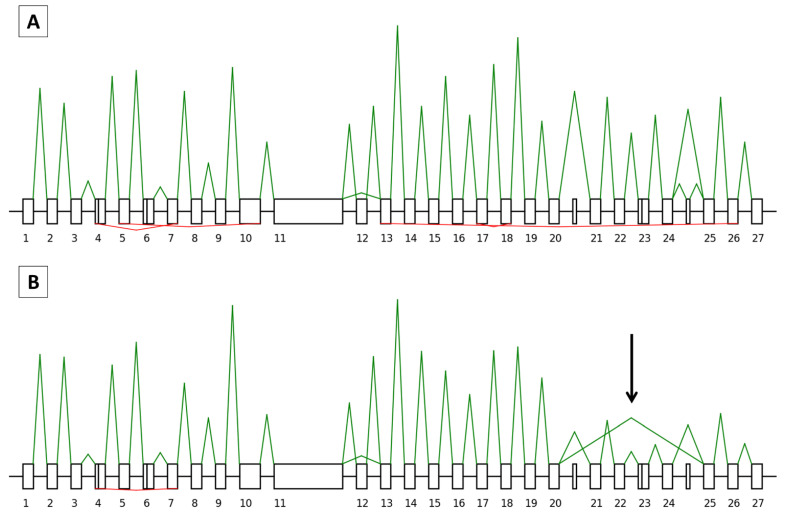
Representation of the *BRCA2* splicing and backsplicing profiles for patient 20X0446, without a known pathogenic *BRCA2* germline variant. Exons are drawn as boxes and are numbered, with the introns represented as lines. A solid line indicates alternative splice sites within the exons. Non-numbered boxes correspond to alternative exons that are not included in the canonical transcript. Peak heights are relative to the number of UMI counts. For each profile, splicing is indicated in green above the boxes and backsplicing in red below the boxes. (**A**) Normal breast tissue and *BRCA2* canonical transcript with the delta 12 isoforms are shown. In red, below, the backsplicing from exons 7 to 4 (circRNA_7-4), 10 to 5 (circRNA_10-5), 18 to 17 (circRNA_18-17), and 26 to 13 (circRNA_26-13) is shown. (**B**) Tumor breast tissue, with the identification of an abnormal skipping from exons 20 to 25, indicated with an arrow, which was not present in the normal tissue.

In all, 66 and 50 junctions, making a total of 37 and 26 isoforms, were correctly identified for *BRCA1* and *BRCA2*, respectively (Figure 3A and Figure 4A). In our FFPE tissue material, SEALigHTS detected exon junctions above 0.26% and 0.15% for *BRCA1* and *BRCA2*, respectively, compared to the RNAseq data from lymphoblastoid cell lines [25]. As a result, the probe design was validated.

SEALigHTS identified the splicing consequences of genomic duplications, deletions, and SNV, i.e., unexpected ligations between exons were evidenced. The *BRCA1* tandem duplication of exon 13 carried by patient 20X0146 was detected thanks to a pseudo circular RNA of exon 13 that is found in normal salpingian and ovarian tissue and in breast tumor tissue. This pseudo circular RNA results from the junction of the probe at the end of exon 13 with the probe at the beginning of the duplicated exon 13 (Figure 3B). Similarly, the *BRCA1* tandem duplication of exons 18 to 20 carried by patient 20X0131 resulted in a pseudo circular RNA joining exon 20 to duplicated exon 18 and was found in the patient’s breast-cancer tissue sample.

Splicing analyses for patients 20X0133, 20X0180, and 20X0198, carrying a germline *BRCA1* deletion encompassing exons 8 to 13, led to an abnormal splice junction from exon 7 to exon 14, found in normal breast (Figure S2A), ovarian, and salpingian tissues. In the breast tumor tissue, this abnormal skipping was found in combination with a decrease in the canonical junctions and an allelic imbalance, suggesting the loss of the wild-type allele in the tumor (Figure S2B). Similarly, splicing analyses in patients 20X0129, 20X0184, and 20X0186, carrying a germline *BRCA1* deletion of exons 3 to 16, led to an abnormal splice junction from exons 2 to 17, evidenced in normal breast, ovarian, and salpingian tissues. In the breast tumor tissue, the combination of abnormal skipping, a decrease in the canonical junctions, and allelic imbalance suggested the loss of the wild-type allele. Lastly, the patient designated as 20X0127, carrying a germline *BRCA1* deletion of exons 18 and 19, showed an abnormal splice junction from exons 17 to 20 in both their normal and tumor breast tissues.

Splicing anomalies caused by three distinct SNV were also detected. The *BRCA1* c.135-1G > C variation, leading to exon 5 skipping, was detected thanks to an abnormal junction of the neighboring exons in normal ovarian and salpingian tissues from patients 20X0172 and 20X0185. No allelic imbalance was observed at the heterozygous SNPs and the relative height of the peaks from the wild-type junctions was similar to the height of the variant junction, in accordance with this in-frame exon skipping (Figure S3). The c.7805G > C variation in the *BRCA2* gene, leading to exon 16 skipping, was detected with a junction between exon 15 and exon 17 in ovarian and salpingian tissue samples from patient 20X0175. The *BRCA2* c.67+3A > G variation (patient 20X0140), leading to exon 2 skipping, was found thanks to an abnormal junction of exons 1 to 3; however, an exon 1 to exon 4 junction was also evidenced in breast tumor tissue. This skipping of exons 2 and 3 has never been described before [25,26] and was not found in any other sample from our series.

In summary, regarding the splicing outcome, SEALigHTS identified all expected splicing consequences of our selected variations, whether they were large deletions, duplications, or point variations. More interesting, as the analyses were conducted in the various tissues of interest, additional and tumor-specific splicing anomalies were detected as a plausible consequence of the second hit.

#### 3.2.2. Backsplicing Analyses

We counted 59 different physiological circular junctions for *BRCA1.* A complete description of the circRNA landscape in breast tissues from the validation set is shown in Table S2. We identified 10 novel ones not listed in RJunBase, comprising: *BRCA1*_circRNA_10-8; 12-6; 16-3; 18-2; 19-17; 20-20; 21-20; 23-22; 23-8p; 5q-3. For *BRCA2*, 23 circular junctions were detected, including 5 novel ones, comprising: *BRCA2*_circRNA_13-12; 26-13; 6q-2; 7-5; 9-9 (Table S3). In this selected set of samples, we found that *BRCA1* produced a higher number of circular RNAs than *BRCA2*.

### 3.3. Research Set

#### 3.3.1. Splicing Study

Analysis of the research set of samples, consisting of pairs of normal and tumor breast tissues from unselected patients, allowed us to find all the physiological mRNA junctions for the *BRCA1* and *BRCA2* genes that were previously identified in the validation set (Figure 3A and Figure 4A). In addition, in one patient, we found an abnormal junction from the *BRCA2* exon 20 to exon 25 in the tumor breast tissue sample that was not present in the corresponding normal tissue. Relative peak height and the absence of allelic imbalance were in accordance with the expected in-frame skipping of exons 21 to 24 (Figure 4B). No other splicing variations were found in the research set.

#### 3.3.2. Backsplicing Study

All circular junctions identified in the validation set were found in the research set and we did not find evidence of other circRNAs. Considering both the tumor and the adjacent tissue samples, the ratio between circRNAs and mRNAs was 1.36 and 0.23 circular junctions per 100 linear junctions for *BRCA1* and *BRCA2*, respectively. In other words, *BRCA1* produced more circRNAs than *BRCA2*. The 20 most frequent *BRCA1* and *BRCA2* circRNAs found in normal and tumor tissues are indicated in Table 1 and Table 2 and the full landscape of circRNAs detected is indicated in Tables S4 and S5. Novel circRNAs are indicated in Table 3 and Table 4.

We identified 59 circRNAs for *BRCA1* and 23 for *BRCA2*. As shown in Table 1 and Table 2 and Tables S4 and S5, their frequency varied whatever the tissue, with almost ubiquitous (found in 32 out of 38 samples) to rare (found in one sample) circRNAs. The diversity of their repertoire ranged from 11.18 % to 0.07% for *BRCA1* and 26.13% to 0.34% for *BRCA2*, and is less pronounced for *BRCA2*, as circRNAs_7-3 and 7-4 accounted for half of the total *BRCA2* circRNAs. Compared to mRNAs, the maximum ratio is 4.73% for BRCA1_circRNA_20-18; in other words, this circRNA made up 4.7% of a mean mRNA junction for *BRCA1* and is found 4.73 times for 100 *BRCA1* mRNA molecules. The same circRNAs were identified in both normal and tumor breast tissue samples. However, individual variations occurred; the most pronounced were: (i) for *BRCA1*, circRNA_23-20, with an increase in relative proportion from 2.71% in the normal tissue to 5.51% in the tumor tissue and circRNA_3-2 with a decrease from 5.42% in the normal tissue to 2.17% in the tumor tissue; (ii) for *BRCA2*, the novel circRNA 26-13 dropped from 4.26% in the normal tissue to 0.67% in the tumor tissue. With the exception of *BRCA1* circRNA_23-20 (see above), lower circRNA/mRNA ratios were observed for all *BRCA1* and *BRCA2* circRNAs, which prompted us to calculate the overall circRNA/mRNA ratio.

**Figure 5:**
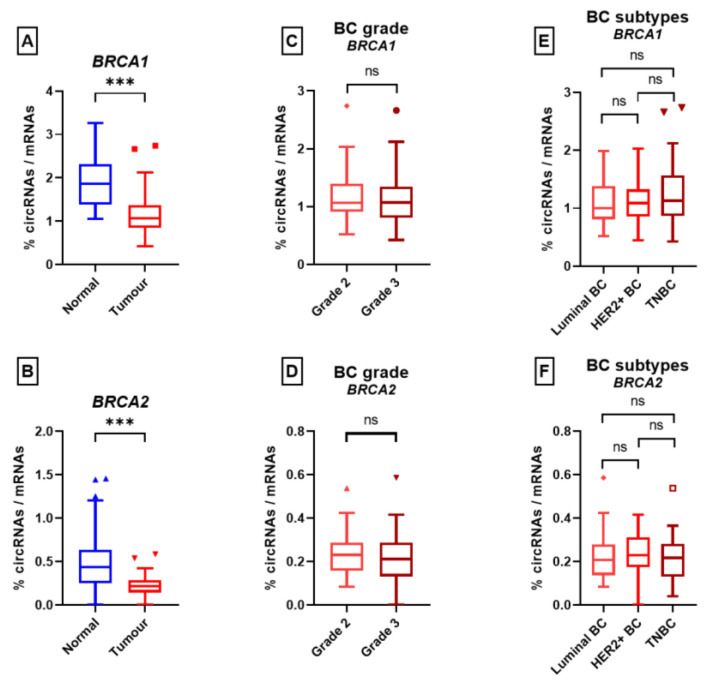
The circular RNA/messenger RNA ratios. Ratios for the normal and tumor tissues are indicated for *BRCA1* (**A**) and *BRCA2* (**B**). Ratios according to histological subtype (luminal, HER2+, and triple-negative breast cancer (TNBC)) for *BRCA1* (**C**) and *BRCA2* (**D**). Ratios according to grade are indicated for *BRCA1* (**E**) and *BRCA2* (**F**). (ns: non-significant; ***: *p* < 0.001, Student’s *t*-test). Triangles, squares and circles represent outliers.

For *BRCA1*, the average ratio of circRNAs/mRNAs was significantly lower in tumor tissue samples compared to normal adjacent mammary tissue samples (1.14 vs. 1.89, *p*-value = 1.6 × 10^−9^) (Figure 5A). This held true for *BRCA2* (0.23 vs. 0.51, *p*-value = 4.4 × 10^−5^) (Figure 5B). We then looked at the histological subtype and for both *BRCA1* and *BRCA2*, no significant difference in the circRNA/mRNA ratio was found for the three BC subtypes (luminal, TNBC, and HER2+) (see Figure 5C,D). Similarly, no significant difference was found between the grade II and grade III samples (*BRCA1*: 1.18 vs. 1.11, *p*-value = 0.47; *BRCA2*: 0.24 vs. 0.2, *p*-value = 0.33) (Figure 5E,F). Overall, these results showed a circRNAs/mRNAs disequilibrium that was not explained by the histological subtype and proliferation status.

## 4. Discussion

Firstly, our study provides the community with an innovative, simple, and high-throughput method for the simultaneous detection of splicing and backsplicing, which has been validated and applied in a series of 354 FFPE samples. Secondly, we have demonstrated a disequilibrium for *BRCA1* and *BRCA2* in the circRNA/mRNA ratio between the tumor and normal breast tissues. These two aspects will be discussed consecutively.

SEALigHTS simultaneously detects mRNAs and circRNAs, thanks to a simple design of probes located at the exon boundaries and following ligation when splicing and backsplicing occur. This assay requires 2 days for the whole procedure, i.e., reverse transcription, hybridization of the probes, ligation, PCR amplification, and analysis on a high-throughput sequencer. No specific materials or platforms are needed; SEALigHTS could be implemented in any laboratory with next-generation sequencing facilities. In our case, the cost per sample was 30 euros (~30 USD). Another strong advantage is the low quality and quantity of input RNA needed (as little as 70 ng), as demonstrated by the variety of tissue samples assayed (breast, ovarian, and salpingian). With a quality threshold of 1500 UMI, more than 90% of the tumor samples tested were successfully analyzed. Low cellularity is obviously a limiting factor and only 37.5% of the normal breast tissue samples provided us with reliable data. This could be explained by the fact that breast tissue has a lower cellularity compared to breast tumor tissue as it is mainly composed of adipocytes, fibrosis, and rare ducts and lobules. Nevertheless, our ability to handle FFPE material suggests that a wide variety of tissues could be robustly assayed. Moreover, the length of the cDNA template is not an issue because probes need a template of only about 60 bases long to be hybridized. Joint analysis of the messenger RNAs and circular RNAs is made easy since no treatment of the sample with exoribonucleases is required and the same bioinformatics tool is used for both transcripts. In theory, SEALigHTS can detect all kinds of splicing events when caused either directly or indirectly. Hence, we successfully identified alternative splicing events and the splicing consequences of SNV, large deletions, and duplications from the validation set, and even demonstrated additional events, e.g., second hits in tumor RNA from both datasets. Although this is a targeted approach, intronic exonization should be detected as a decrease in the number of ligations is expected at the corresponding exon junctions. Allelic imbalance is detected by using exonic SNPs, and, more generally, we believe that gene expression could be calculated from the splicing data as RT-MLPseq and LD-RT-PCR provided robust gene expression measurements [21,22].

SEALigHTS deciphered the *BRCA1* and *BRCA2* backsplicing landscape in normal and tumor breast tissues and identified not only the already described circRNAs but also 10 and 5 novel ones for *BRCA1* and *BRCA2*, respectively. Previous literature on breast cancer suggested that specific circular RNAs would be deregulated between normal and tumor tissues [27,28]; however, despite the fact that *BRCA1* and *BRCA2* are master genes of homologous recombination and breast cancer predisposition, our study is the first to address the question of their circular RNAs in breast cancer. We first evidenced a larger number of circRNAs in *BRCA1* than in *BRCA2*, in accordance with RJunBase, with a similar qualitative repertoire between normal and tumor tissues. We did show individual circRNA variations, e.g., *BRCA1*_circRNA_3-2 and *BRCA2*_circRNA_26-13, which could be linked to the tumor process, but these findings should be replicated on a larger series to draw definite conclusions. Hence, if the functional importance of *BRCA1*_circRNA_3-2 could be speculated because *BRCA1* exon 2 contains the wild-type translation initiation sequence, a putative functional mechanism for *BRCA2* circRNA_26-13, which nearly disappears from tumor samples, remains unknown. Moreover, the relative proportion of each circRNA remains low, compared to its mRNA counterpart, and does not exceed 5%.

On the other hand, we demonstrated, for the first time, a decrease of 40% and 55% in the circRNA/mRNA ratio for *BRCA1* and *BRCA2*, respectively, in breast cancer tissues compared to normal adjacent tissues. This disequilibrium can be viewed as a cause or a consequence of tumorigenesis. circRNAs are usually described as miRNA sponges, although this general scenario may actually be extrapolated from a few cases [29]. Regardless, this sponge mechanism is not an obvious explanation here because miRNAs are known to foster or repress proliferation, and *BRCA* circRNAs should thus only target proliferative miRNAs. Another explanation can be found in the mandatory balance between circRNAs and mRNAs. Assuming that circRNAs biogenesis competes with mRNAs, our findings may at first sound counterintuitive as breast tumor tissues would be less buffered by circRNAs. It is actually tempting to speculate that this decrease reflects an attempt by the cell to counter the tumor process by lowering the number of circular RNAs, in order to maintain a sufficient level of messenger RNAs. Our results echo the recent description of a PTEN circular RNA, circPTEN1, acting as a tumor suppressor that is downregulated in the tumor tissues of colorectal cancer [30], as well as a previous RNA-seq study showing that circRNAs were globally reduced in tumor tissues from colorectal cancer patients, compared to matched normal tissues [31], suggesting a negative correlation of global circRNA abundance and proliferation. However, this result is not supported by our own findings as no significant difference was found between proliferative triple-negative subtype tumors and the less proliferative luminal subtype tumors. The question remains open as to whether our findings are restricted to *BRCA1* and *BRCA2* or if they represent a small part of a global reduction of circRNAs in breast cancer. It cannot be rejected that this reduction in circular RNA abundance possibly reflects an as-yet unknown tumorigenic hallmark of tissue, as global splicing dysregulation is already known in cancer.

## 5. Conclusions

Overall, we developed an innovative assay method to study backsplicing and linear splicing, which can be easily translated into diagnostics. We then deciphered the landscape of *BRCA* circRNAs, described novel ones, and demonstrated that the ratio between backsplicing and linear splicing in *BRCA1* and *BRCA2* is not constant but evolves according to the cell environment. Finally, we suggested that a disequilibrium between *BRCA1/2* circular and messenger RNAs in favor of mRNA could reflect a tentative adaptation to tumorigenesis. This study should now be pursued further using a larger series with other genes from the homologous recombination pathway.

## Figures and Tables

**Table 1:** The 20 most represented and novel *BRCA1* circular RNAs in the normal and tumor breast tissues of the research set. Twenty-one circRNAs are listed as circRNA22-15 is among the 20 most represented in the tumor tissue samples, but not in the normal tissue samples. Conversely, 5q-3 is among the 20 most commonly represented circRNAs in the normal tissue samples, but not in the tumor tissue samples. The novel circRNAs are indicated in bold.

	*BRCA1* Circular RNAs NORMAL			*BRCA1* Circular RNAs TUMOR		
Circular RNA Name	Relative Proportion of *BRCA1* circRNAs (%)	Frequency (%) (n = 38)	Ratio of circRNA/mRNA (%)	Relative Proportion to *BRCA1* circRNAs (%)	Frequency (%) (n = 90)	Ratio of circRNA/mRNA (%)
BRCA1_circRNA_20-18	10.94	84.21 (32)	4.73	11.18	92.22 (83)	2.57
BRCA1_circRNA_7-6	10.83	89.47 (34)	4.68	7.93	87.78 (79)	1.82
BRCA1_circRNA_17-15	10.31	92.11 (35)	4.46	9.49	92.22 (83)	2.18
BRCA1_circRNA_19-18	7.19	78.95 (30)	3.11	5.80	84.44 (76)	1.33
BRCA1_circRNA_3-2	5.42	44.74 (17)	2.34	2.17	46.67 (42)	0.50
BRCA1_circRNA_10-2	5.00	68.42 (26)	2.16	3.78	72.22 (65)	0.87
BRCA1_circRNA_22-20	3.65	50 (19)	1.58	4.81	87.78 (79)	1.11
BRCA1_circRNA_10-6	3.33	44.74 (17)	1.44	2.66	63.33 (57)	0.61
BRCA1_circRNA_12-11	3.33	55.26 (21)	1.44	5.25	84.44 (76)	1.21
BRCA1_circRNA_23-18	3.33	55.26 (21)	1.44	5.15	81.11 (73)	1.18
BRCA1_circRNA_19-15	3.23	52.63 (20)	1.40	2.59	72.22 (65)	0.60
BRCA1_circRNA_8-6	2.81	55.26 (21)	1.22	2.08	56.67 (51)	0.48
BRCA1_circRNA_23-20	2.71	42.11 (16)	1.17	5.51	81.11 (73)	1.27
BRCA1_circRNA_21-18	2.40	34.21 (13)	1.04	2.68	63.33 (57)	0.62
BRCA1_circRNA_8-3	2.08	42.11 (16)	0.90	2.91	76.67 (69)	0.67
**BRCA1_circRNA_5q-3**	1.67	13.16 (5)	0.72	0.71	22.22 (20)	0.16
BRCA1_circRNA_22-18	1.56	31.58 (12)	0.68	2.37	61.11 (55)	0.54
BRCA1_circRNA_23-15	1.46	28.95 (11)	0.63	1.24	43.33 (39)	0.28
BRCA1_circRNA_7-5	1.35	28.95 (11)	0.59	1.69	41.11 (37)	0.39
BRCA1_circRNA_20-15	1.15	18.42 (7)	0.50	1.75	55.56 (50)	0.40
BRCA1_circRNA_22-15	1.04	26.32 (10)	0.45	1.69	57.78 (52)	0.39

**Table 2:** The 20 most represented and novel *BRCA2* circular RNAs in the normal and tumor breast tissue samples of the research set. Twenty-one circRNAs are listed, as circRNA22-15 is among the 20 most represented in the tumor tissue samples, but not in the normal tissue samples. Conversely, 5q-3 is among the 20 most commonly represented circRNAs in the normal tissue, but not in the tumor tissue samples. The novel circRNAs are indicated in bold.

	*BRCA2* Circular RNAs NORMAL			*BRCA2* Circular RNAs TUMOR		
Circular RNA Name	Relative Proportion to *BRCA1* circRNAs (%)	Frequency (%) (n = 38)	Ratio of circRNA/mRNA (%)	Relative Proportion to *BRCA1* circRNAs (%)	Frequency (%) (n = 90)	Ratio of circRNA/mRNA (%)
BRCA2_circRNA_7-3	22.34	50 (19)	2.76	26.13	76.67 (69)	1.48
BRCA2_circRNA_7-4	19.68	55.26 (21)	2.43	21.18	76.67 (69)	1.20
BRCA2_circRNA_18-17	12.23	44.74 (17)	1.51	12.61	61.11 (55)	0.71
**BRCA2_circRNA_7-5**	6.91	21.05 (8)	0.85	5.97	48.89 (44)	0.34
**BRCA2_circRNA_9-9**	4.79	21.05 (8)	0.59	2.10	24.44 (22)	0.12
**BRCA2_circRNA_26-13**	4.26	21.05 (8)	0.53	0.67	8.89 (8)	0.04
BRCA2_circRNA_21-20	3.72	7.89 (3)	0.46	1.51	18.89 (17)	0.09
BRCA2_circRNA_13-11	3.19	13.16 (5)	0.39	7.39	47.78 (43)	0.42
BRCA2_circRNA_4-3	3.19	13.16 (5)	0.39	3.61	32.22 (29)	0.20
BRCA2_circRNA_14-13	2.66	13.16 (5)	0.33	1.43	14.44 (13)	0.08
BRCA2_circRNA_10-4	2.13	7.89 (3)	0.26	1.01	10 (9)	0.06
**BRCA2_circRNA_13-12**	2.13	5.26 (2)	0.26	0.76	10 (9)	0.04
**BRCA2_circRNA_6q-2**	2.13	10.53 (4)	0.26	0.76	10 (9)	0.04
BRCA2_circRNA_10-5	1.60	7.89 (3)	0.20	0.67	7.78 (7)	0.04
BRCA2_circRNA_10-8	1.60	7.89 (3)	0.20	1.43	17.78 (16)	0.08
BRCA2_circRNA_11-11	1.60	7.89 (3)	0.20	1.18	13.33 (12)	0.07
BRCA2_circRNA_10-3	1.06	5.26 (2)	0.13	1.01	12.22 (11)	0.06
BRCA2_circRNA_19-17	1.06	5.26 (2)	0.13	3.28	32.22 (29)	0.19
BRCA2_circRNA_24-19	1.06	5.26 (2)	0.13	2.10	20 (18)	0.12
BRCA2_circRNA_24-22	1.06	5.26 (2)	0.13	1.76	16.67 (15)	0.10
BRCA2_circRNA_24-21	0.53	2.63 (1)	0.07	2.44	28.89 (26)	0.14

**Table 3:** Novel *BRCA1* circular RNAs found in the normal and tumor breast tissue samples of the research set.

	*BRCA1* Circular RNAs NORMAL			*BRCA1* Circular RNAs TUMOR		
Circular RNA Name	Relative proportion to *BRCA1* circRNAs (%)	Frequency (%) (n = 38)	Ratio of circRNA/mRNA (%)	Relative Proportion to *BRCA1* circRNAs (%)	Frequency (%) (n = 90)	Ratio of circRNA/mRNA (%)
**BRCA1_circRNA_5q-3**	1.67	13.16 (5)	0.72	0.71	22.22 (20)	0.16
**BRCA1_circRNA_23-8p**	1.15	23.68 (9)	0.50	0.76	40 (36)	0.18
**BRCA1_circRNA_19-17**	1.04	21.05 (8)	0.45	0.85	38.89 (35)	0.19
**BRCA1_circRNA_18-2**	0.63	13.16 (5)	0.27	0.25	14.44 (13)	0.06
**BRCA1_circRNA_16-3**	0.52	13.16 (5)	0.23	0.07	4.44 (4)	0.02
**BRCA1_circRNA_23-22**	0.42	10.53 (4)	0.18	0.10	6.67 (6)	0.02
**BRCA1_circRNA_10-8**	0.31	7.89 (3)	0.14	0.66	27.78 (25)	0.15
**BRCA1_circRNA_20-20**	0.31	5.26 (2)	0.14	0.41	11.11 (10)	0.09
**BRCA1_circRNA_12-6**	0.10	2.63 (1)	0.05	0.19	12.22 (11)	0.04
**BRCA1_circRNA_21-20**	0.10	2.63 (1)	0.05	0.10	6.67 (6)	0.02

**Table 4:** Novel *BRCA2* circular RNAs found in the normal and tumor breast tissue samples of the research set.

	*BRCA2* Circular RNAs NORMAL			*BRCA2* Circular RNAs TUMOR		
Circular RNA Name	Relative Proportion to *BRCA1* circRNAs (%)	Frequency (%) (n = 38)	Ratio circRNA/mRNA (%)	Relative Proportion to *BRCA1* circRNAs (%)	Frequency (%) (n = 90)	Ratio circRNA/mRNA (%)
**BRCA2_circRNA_7-5**	6.91	21.05 (8)	0.85	5.97	48.89 (44)	0.34
**BRCA2_circRNA_9-9**	4.79	21.05 (8)	0.59	2.10	24.44 (22)	0.12
**BRCA2_circRNA_26-13**	4.26	21.05 (8)	0.53	0.67	8.89 (8)	0.04
**BRCA2_circRNA_13-12**	2.13	5.26 (2)	0.26	0.76	10 (9)	0.04
**BRCA2_circRNA_6q-2**	2.13	10.53 (4)	0.26	0.76	10 (9)	0.04

## Data Availability

Part of the data presented in this study are available on request from the corresponding author, due to the large number of raw files generated.

## Supplementary Materials

The following supporting information can be downloaded at: https://www.mdpi.com/article/10.3390/cancers15072176/s1, Figure S1: Linear splicing and backsplicing processes; Figure S2: Representation of *BRCA1* splicing and backsplicing mammary tissue profiles; Figure S3: Representation of *BRCA1* splicing and backsplicing salpingian tissue profiles; Table S1: *BRCA1* and *BRCA2* germline splice variations from the validation set; Table S2: All the *BRCA1* and *BRCA2* circular RNAs in the normal and tumor breast tissues of the validation set; Table S3: The novel *BRCA1* and *BRCA2* circular RNAs in the breast tissues of the validation set; Table S4: All the *BRCA1* circular RNAs in the normal and tumor breast tissues of the research set; Table S5: All the *BRCA2* circular RNAs in the normal and tumor breast tissues of the research set.
