# Supplementary figures and images for: Disequilibrium between BRCA1 and BRCA2 Circular and Messenger RNAs Plays a Role in Breast Cancer
Source: Cancers (Basel). 2023 Apr 6;15(7):2176. doi: 10.3390/cancers15072176 (PMC10093293; doi:10.3390/cancers15072176)

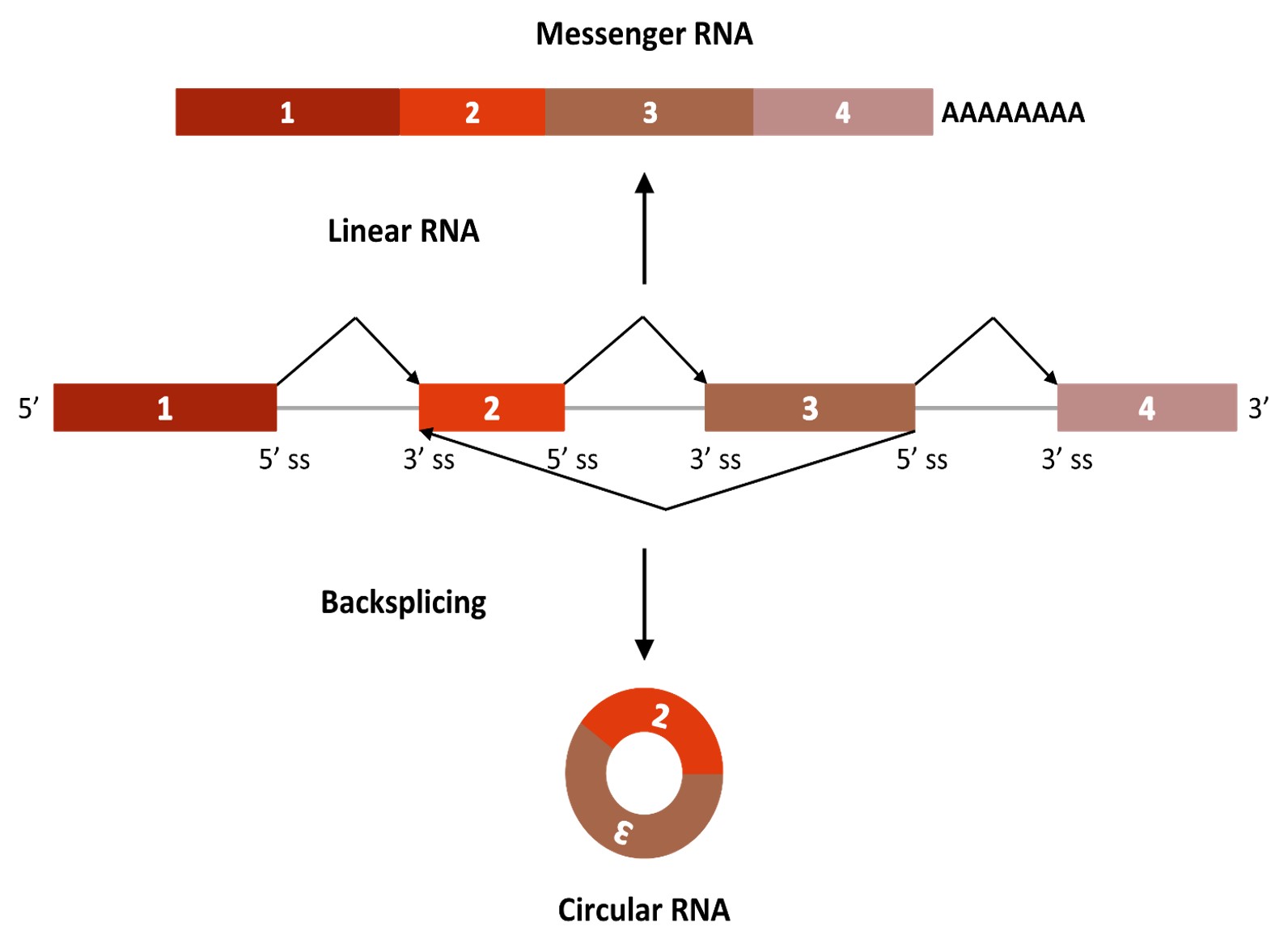

Supplement: Supplementary file 1 [file cancers-15-02176-s001.zip › Supplementary Figure S1.jpg]

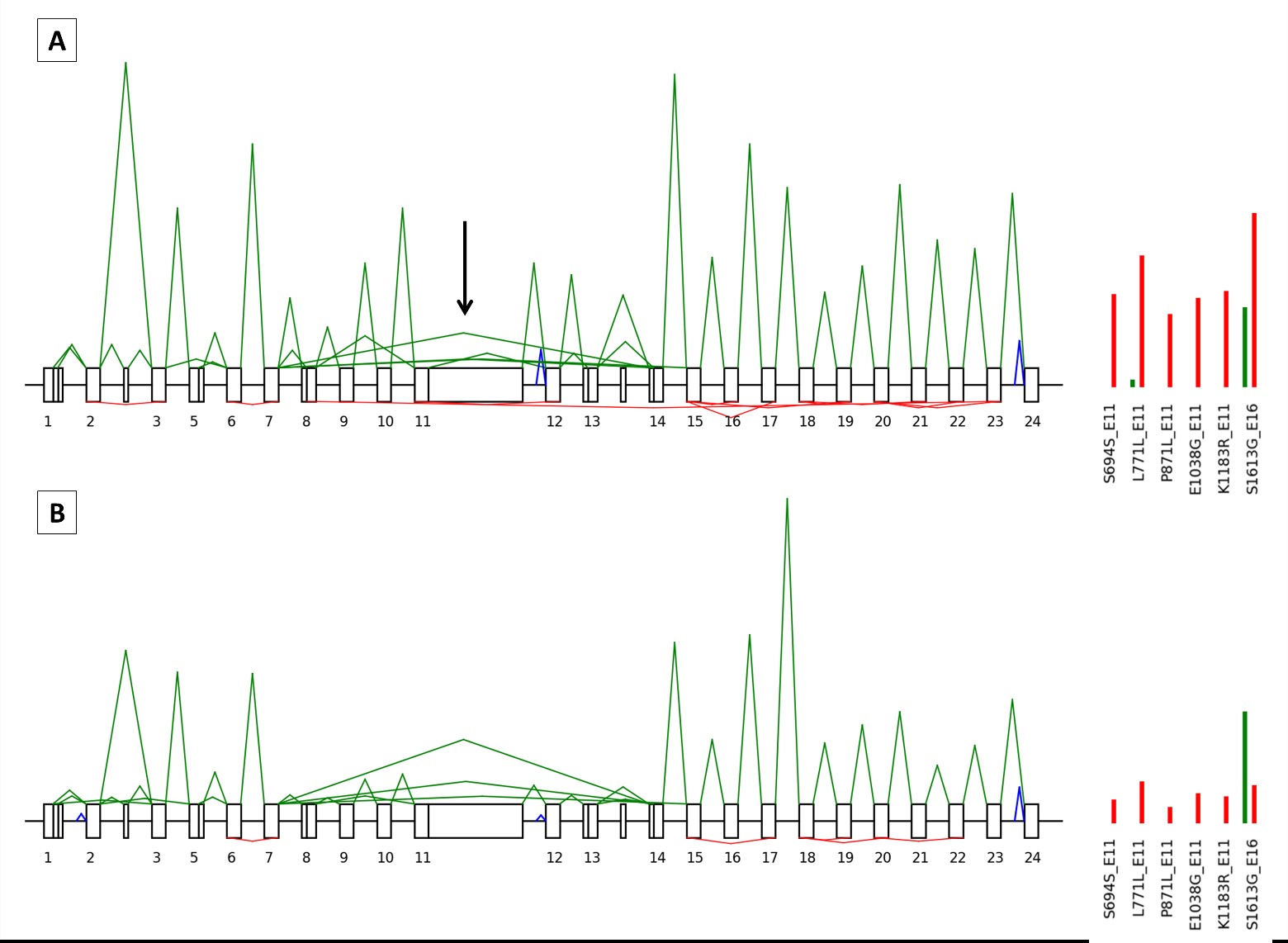

Supplement: Supplementary file 1 [file cancers-15-02176-s001.zip › Supplementary Figure S2.jpg]

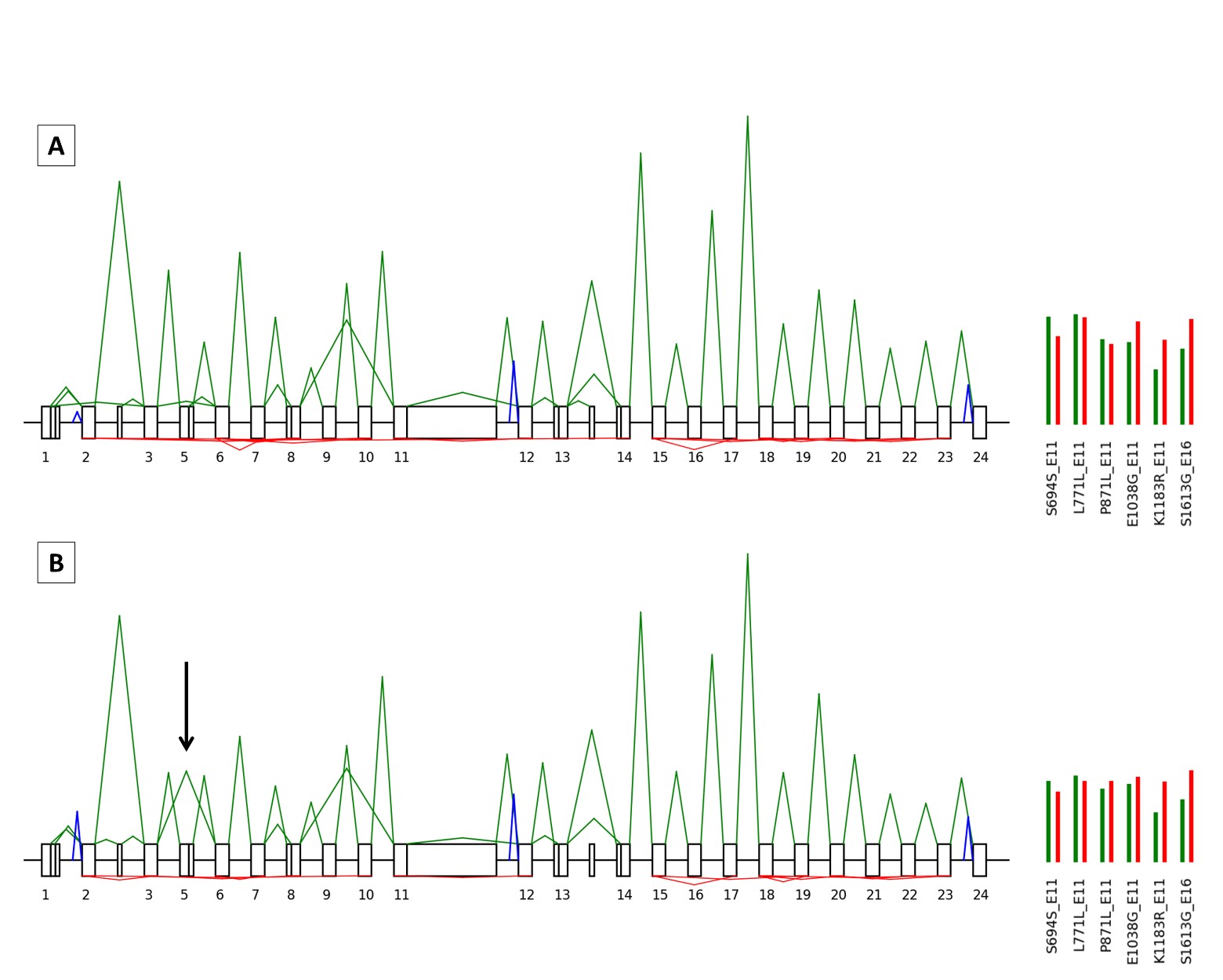

Supplement: Supplementary file 1 [file cancers-15-02176-s001.zip › Supplementary Figure S3.jpg]
